# Metabolic Syndrome Among Inpatients With Schizophrenia: Prevalence and Clinical Correlates

**DOI:** 10.31083/AP56362

**Published:** 2026-10-16

**Authors:** Mao Tang, She-Hui Chang, Hang Qian, Chang-Yong Zhang, Bao-Liang Zhong

**Affiliations:** ^1^Department of Psychiatry, Affiliated Wuhan Mental Health Center, Tongji Medical College of Huazhong University of Science and Technology, 430012 Wuhan, Hubei, China; ^2^Research Center for Psychological and Health Sciences, China University of Geosciences, 430074 Wuhan, Hubei, China; ^3^Department of Psychiatry, Wuhan Wudong Hospital (Wuhan Second Mental Hospital), 430084 Wuhan, Hubei, China; ^4^Department of Psychiatry, Wuhan Mental Health Center, 430012 Wuhan, Hubei, China; ^5^Hubei Clinical Research Center for Whole-Course Management of Late-Life Mental Disorders, 430012 Wuhan, Hubei, China

**Keywords:** metabolic syndrome, schizophrenia, antipsychotic, depressive symptoms, inpatients

## Abstract

**Background:**

Metabolic syndrome (MetS) is a major physical comorbidity in schizophrenia and is associated with increased risks of cardiovascular disease, diabetes, and poor prognosis. This study investigated the prevalence of MetS and its clinical correlates among Chinese inpatients with schizophrenia, with particular attention to the metabolic-risk profiles of antipsychotic regimens.

**Methods:**

This multicenter cross-sectional study included 1024 inpatients with schizophrenia from two tertiary psychiatric hospitals in Central China. MetS was diagnosed according to the criteria of the Chinese Diabetes Society. Sociodemographic, clinical, medication, physical examination, and laboratory data were collected. Antipsychotic regimens were classified as non-high-risk, high-risk, or high-risk-plus-low-risk combinations. Multivariable logistic regression was used to identify factors independently associated with MetS.

**Results:**

Overall, 398 of 1024 patients met the criteria for MetS, yielding a prevalence of 38.9% (95% confidence interval (CI), 35.9%–41.9%). Compared with patients without MetS, those with MetS were more likely to be male, older, have a higher body mass index, have a longer illness duration, exhibit depressive symptoms, report a history of smoking and alcohol use, and have greater illness severity. In the multivariable analysis, male sex (adjusted odds ratio [aOR] = 1.65, 95% CI, 1.21–2.25), older age (aOR = 1.02, 95% CI, 1.00–1.03), illness duration >5 years (aOR = 1.55, 95% CI, 1.14–2.13), Calgary Depression Scale for Schizophrenia (CDSS)-defined depressive symptoms (aOR = 2.71, 95% CI, 1.87–3.92), and high-risk antipsychotic regimens (aOR = 1.54, 95% CI, 1.15–2.06) were independently associated with MetS. High-risk-plus-low-risk combination regimens and chlorpromazine-equivalent (CPZ-eq) dose were not significantly associated with MetS.

**Conclusions:**

MetS is highly prevalent in this sample of Chinese inpatients with schizophrenia. The findings highlight the associations of both patient-level characteristics and antipsychotic regimen composition with MetS, whereas the current CPZ-equivalent dose showed no significant association. Enhanced metabolic monitoring and individualized treatment strategies are warranted in inpatient schizophrenia care.

## Main Points

1. The prevalence of metabolic syndrome among inpatients with schizophrenia was 38.9%.

2. Male sex, older age, longer illness duration, depressive symptoms, and high-metabolic-risk antipsychotic regimens were independently associated with metabolic syndrome.

3. Patients receiving high- and low-metabolic-risk combination regimens showed no significantly higher odds of metabolic syndrome than those receiving non-high-risk regimens, whereas high-risk regimens were associated with higher odds of Metabolic syndrome (MetS).

4. These findings support incorporating the metabolic profiles of individual antipsychotics into treatment selection and cardiometabolic monitoring in patients with schizophrenia.

## 1. Introduction

Among the physical comorbidities of schizophrenia, metabolic syndrome (MetS) is of particular clinical importance because it is common and closely linked to cardiovascular disease, type 2 diabetes, and poor overall prognosis [1]. In people with schizophrenia, MetS refers to the clustering of central obesity, hypertension, dysglycemia, hypertriglyceridemia, and reduced high-density lipoprotein cholesterol, and it has become a major target for integrated psychiatric and medical care [2,3].

Recent evidence indicates that the burden of MetS in schizophrenia is consistently high. A classic meta-analysis estimated that the prevalence of MetS in schizophrenia and related disorders was approximately one-third worldwide [3], while a more recent global meta-analysis reported an even higher pooled prevalence of 41.3% [4]. In China, a recent meta-analysis of 73 studies involving 34,655 patients reported a pooled prevalence of 31.4% [5]. In addition, a large multicenter study of hospitalized patients with schizophrenia in China reported a prevalence of 34.9% using the Chinese Diabetes Society criteria [6]. Together, these findings suggest that MetS is a highly prevalent and clinically significant comorbidity in schizophrenia.

Existing research further suggests that MetS in schizophrenia is multifactorial. Prior studies have examined a broad range of associated factors, including sociodemographic characteristics, lifestyle-related exposures, and illness-related variables [7]. Across recent Chinese evidence, older age, smoking, higher body mass index, and longer illness duration have emerged as relatively consistent correlates, whereas associations with sex, education, and marital status have varied across samples [5,6]. In addition to these factors, depressive symptoms deserve particular attention. Depression is common across the course of schizophrenia, and emerging evidence suggests that depressive morbidity may be associated with greater metabolic burden in this population. In a recent study of remitted schizophrenia, MetS remained significantly associated with comorbid major depressive disorder after adjustment for key demographic and clinical variables, highlighting the need to consider depressive symptoms alongside cardiometabolic risk [8]. Although the mechanisms remain incompletely understood, shared behavioral, inflammatory, neuroendocrine, and metabolic pathways have been proposed [2,9].

Antipsychotic treatment is one of the most clinically modifiable determinants of metabolic risk, but the relationship between prescribing strategy and MetS remains unsettled. Comparative evidence has consistently shown marked differences in metabolic liability across agents, with clozapine and olanzapine generally carrying the highest risk, whereas aripiprazole, lurasidone, and ziprasidone tend to have more favorable metabolic profiles [10]. At the same time, treatment guidelines and major reviews continue to prioritize antipsychotic monotherapy whenever possible, because polypharmacy is often associated with greater treatment complexity and a higher burden of adverse effects [11,12]. However, the evidence regarding whether antipsychotic polypharmacy itself confers a higher risk of MetS than monotherapy remains conflicting [12].

The question becomes even more complex when dose is considered. Recent dose-response meta-analytic evidence suggests that metabolic adverse effects are not uniformly linear across antipsychotics; rather, the shapes of the dose-response curves differ by drug, with plateau, ascending, and bell-shaped patterns all being observed [13]. Likewise, although adjunctive aripiprazole has shown potential benefits for glycolipid metabolism in some higher-risk regimens, the current evidence remains limited and is not yet sufficient to support firm conclusions across all combination strategies [14,15]. These inconsistencies suggest that classifying exposure simply as monotherapy versus polypharmacy, or relying on total chlorpromazine-equivalent (CPZ-eq) dose alone, may be too crude to capture the true metabolic impact of different prescribing patterns. More granular evidence is needed on combination regimens composed of antipsychotics with different intrinsic metabolic liabilities.

Against this background, further evidence is needed on the prevalence and correlates of MetS among Chinese inpatients with schizophrenia, particularly in real-world settings where chronic illness, reduced activity, and cumulative medication exposure may heighten cardiometabolic vulnerability. Therefore, the present study investigated the prevalence of MetS and its associated factors among inpatients with schizophrenia in two tertiary psychiatric hospitals in Central China, with particular attention to the metabolic risk profile of antipsychotic prescriptions. By examining not only overall antipsychotic exposure but also combinations of antipsychotics with different metabolic risk levels, this study aimed to provide evidence that may help optimize antipsychotic selection and strengthen cardiometabolic monitoring in routine clinical practice.

## 2. Materials and Methods

### 2.1 Study Design, Setting, and Participants

This multicenter cross-sectional study was conducted at Wuhan Mental Health Center and Wuhan Wudong Hospital (Wuhan Second Mental Hospital) between March 1, 2024, and October 1, 2025, the two largest municipal public psychiatric specialty hospitals in Wuhan, Central China. In 2025, the two hospitals had a combined total of 1950 inpatient beds, 25,272 inpatient discharges, and 524,280 outpatient visits. Together, they served as major public providers of mental health care for Wuhan, a city with approximately 13.86 million permanent residents [16].

Hospitalized patients were consecutively screened for eligibility from the psychiatric wards of the two hospitals during the study period. Patients were eligible if they: (1) had a diagnosis of schizophrenia established by attending psychiatrists according to the International Classification of Diseases, Tenth Revision (ICD-10); (2) were aged 18 years or older.

Patients were excluded if they: (1) had an organic brain disorder or an unstable or severe medical condition that precluded participation; (2) had a comorbid psychoactive substance use disorder; (3) had an intellectual disability; (4) were unable to complete the study assessment because of severe agitation, acute suicidal risk, or another acute psychiatric condition; or (5) were pregnant or breastfeeding.

A total of 1406 inpatients with schizophrenia were identified from the study database. For the present analysis, 382 patients were excluded because of incomplete data required for MetS ascertainment (n = 130), missing antipsychotic treatment data (n = 163), or missing demographic information (n = 89). The final analytic sample therefore comprised 1024 patients (72.8% of the screened sample; Fig. 1).

**Fig. 1.**
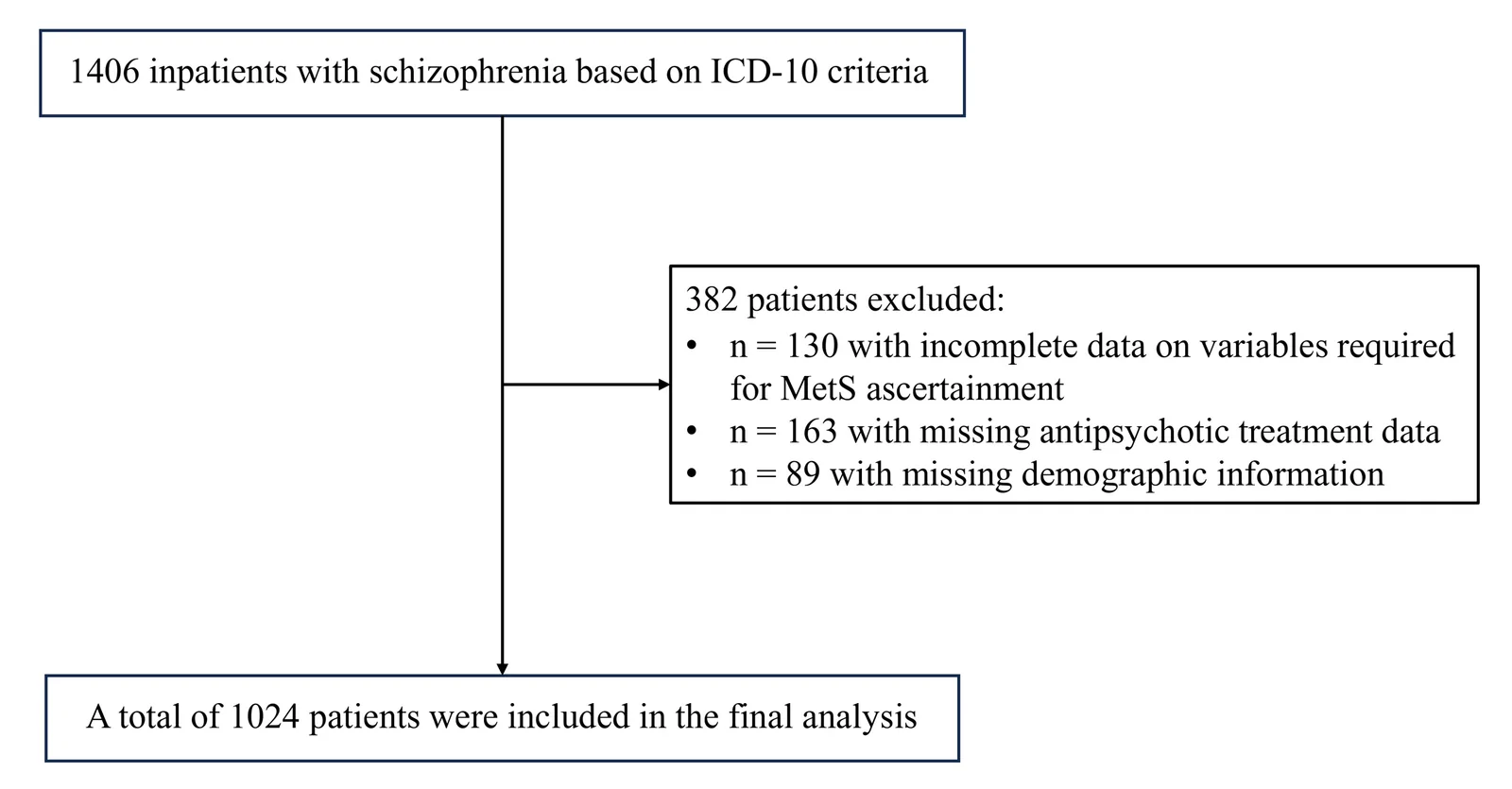
**Flowchart of participant selection**. ICD-10, International Classification of Diseases, Tenth Revision; MetS, Metabolic syndrome.

The study protocol was approved by the Medical Ethics Committee of Wuhan Mental Health Center, written informed consent was obtained from the patients and their family members, and verbal informed consent was obtained from the treating psychiatrists.

### 2.2 Clinical and Medication Assessments

Sociodemographic and clinical data were collected from medical records and face-to-face assessments, including age, sex, education level, marital status, employment status, smoking history, alcohol use history, family history of psychiatric disorders, age at illness onset, duration of illness, depressive symptoms, current antipsychotic treatment, and daily antipsychotic dose.

At study enrollment, depressive symptoms and overall illness severity were assessed by psychiatrists who had received standardized training in scale administration. Depressive symptoms were evaluated using the validated Chinese version of the Calgary Depression Scale for Schizophrenia (CDSS) [17], a 9-item clinician-rated scale with a total score ranging from 0 to 27; a score ≥5 was used to indicate clinically significant depressive symptoms [18]. Overall illness severity was assessed using the Clinical Global Impression–Severity (CGI-S), rated from 1 (normal, not at all ill) to 7 (among the most extremely ill), with scores ≥4 categorized as moderate-to-severe illness [19]. Formal inter-rater reliability was not quantified in the present study.

To account for differences in current overall antipsychotic dose, CPZ-eq daily dose was included as a covariate in the analyses. For descriptive and regression analyses, CPZ-eq dose was further categorized into three predefined analytical groups: low (≤200 mg/day), medium (201–400 mg/day), and high (>400 mg/day) [20,21].

### 2.3 Physical Examination and Laboratory Measurements

Trained nurses conducted physical examinations in accordance with standardized protocols. Waist circumference was measured to the nearest 0.1 cm using a non-elastic tape at the midpoint between the lower rib margin and the iliac crest. Height and body weight were measured, and body mass index (BMI) was calculated as weight in kilograms divided by height in meters squared. Blood pressure was measured on the right arm in the seated position using a regularly calibrated medical-grade electronic sphygmomanometer (Omron Healthcare Co., Ltd., Kyoto, Japan) after the participant had rested for at least 5 minutes. Each physical parameter was measured twice, and the average value was used in the analyses.

Laboratory assessments included fasting triglycerides (TG), high-density lipoprotein cholesterol (HDL-C), and fasting blood glucose (FBG). All participants fasted for at least 8 hours before blood sampling. Fasting venous blood samples were collected by nurses on the following morning and analyzed in the hospital clinical laboratory using standard enzymatic colorimetric methods on automated analyzers (Shenzhen Mindray Bio-Medical Electronics Co., Ltd., Shenzhen, China). In cases of markedly abnormal results, retesting or repeat sampling was performed in accordance with standard operating procedures.

### 2.4 Definition of Antipsychotic Metabolic-Risk Profiles and MetS

To better characterize the metabolic-risk profile of antipsychotic treatment, the 10 antipsychotics used in the present sample were first classified according to their established metabolic liability. Clozapine and olanzapine were classified as high-metabolic-risk antipsychotics; quetiapine, risperidone, and paliperidone as moderate-metabolic-risk antipsychotics; and aripiprazole, lurasidone, ziprasidone, perospirone, and amisulpride as low-metabolic-risk antipsychotics, based on evidence from comparative studies and expert consensus [10,22,23,24]. At the regimen level, current antipsychotic prescriptions were operationally categorized into three mutually exclusive groups: non-high-risk regimens, high-risk regimens, and high-risk-plus-low-risk combination regimens. Non-high-risk regimens contained no high-risk antipsychotic and could therefore consist of moderate-risk and/or low-risk agents. High-risk regimens contained at least one high-risk antipsychotic but no low-risk agent, whereas high-risk-plus-low-risk combination regimens contained at least one high-risk and at least one low-risk antipsychotic. Detailed distributions and daily doses of individual antipsychotics are provided in **Supplementary Table 1**, and the specific current antipsychotic regimens are shown in **Supplementary Table 2**.

The high-risk-plus-low-risk group was analyzed separately because the metabolic consequences of antipsychotic polypharmacy may depend on regimen composition rather than simply on the number of agents used, and previous evidence regarding the association between polypharmacy and MetS has been heterogeneous [11,12,25]. In addition, lower-metabolic-risk agents, particularly aripiprazole, have been used adjunctively with higher-risk antipsychotics and may have favorable metabolic effects in selected settings, although the evidence remains insufficient to establish such combinations as metabolically protective [14]. Accordingly, this category was defined as a practice-informed, hypothesis-generating classification rather than an established low-risk treatment.

MetS was diagnosed according to the criteria recommended by the Chinese Diabetes Society of the Chinese Medical Association [26]. Participants were considered to have MetS if they met at least three of the following five criteria: (1) abdominal obesity, defined as a waist circumference ≥90 cm in men and ≥85 cm in women; (2) hyperglycemia, defined as FBG ≥6.1 mmol/L and/or previously diagnosed diabetes under treatment; (3) hypertension, defined as blood pressure ≥130/85 mmHg and/or previously diagnosed hypertension under treatment; (4) TG ≥1.70 mmol/L; and (5) HDL-C <1.04 mmol/L.

### 2.5 Other Covariates

Other covariates included demographic characteristics, lifestyle factors, and illness-related variables. Education level was categorized into three groups: primary school or below, middle/high school, and college or above. Marital status was categorized as unmarried, married, and divorced/widowed. Employment status was categorized as employed versus unemployed/retired.

Smoking history and alcohol use history were each recorded as never, former, or current. Because the number of former users was limited, former and current users were combined into ever-use categories for analysis.

For descriptive subgroup analyses, illness duration was further categorized into early-course (≤5 years) and late-course (>5 years) illness. Age at onset was dichotomized as <18 years and ≥18 years, using the conventional age threshold for early-onset schizophrenia [27].

### 2.6 Statistical Analysis

All statistical analyses were performed using R software (version 4.3.1; R Foundation for Statistical Computing, Vienna, Austria). Descriptive statistics were used to summarize demographic and clinical characteristics. Categorical variables were presented as frequencies and percentages and were compared using the chi-square test. Continuous variables with approximately normal distributions were expressed as mean ± standard deviation (SD) and were compared using the independent-samples *t* test. Variables with skewed distributions were summarized as medians and interquartile ranges (IQRs) and were compared using the Mann–Whitney U test.

To examine factors associated with MetS in inpatients with schizophrenia, a multivariable logistic regression model was constructed with MetS status (MetS vs. no MetS) as the dependent variable. Covariates included in the multivariable model were selected based on clinical relevance and evidence from previous literature and included age, sex, age at illness onset, illness duration, education level, marital status, depressive symptoms, illness severity, ever smoking, ever alcohol use, CPZ-equivalent dose, and antipsychotic metabolic-risk profile. BMI was not included in the multivariable model because it is an adiposity measure closely related to waist circumference, a defining component of MetS, and adjustment for BMI could lead to overadjustment. Multicollinearity among covariates was assessed using variance inflation factors (VIFs), with no evidence of problematic multicollinearity (maximum VIF = 1.99). Potential nonlinear associations of age and illness duration with MetS were assessed using 3-degree-of-freedom natural spline models. In a sensitivity analysis, illness duration was additionally modeled as a continuous variable, with effect estimates expressed per 5-year increase. The strength of association was estimated using adjusted odds ratios (aORs) and 95% confidence intervals (95% CIs). As a sensitivity analysis, modified Poisson regression with robust variance estimation was performed to estimate adjusted prevalence ratios (aPRs). All tests were two-sided, and *p* < 0.05 was considered statistically significant.

## 3. Results

### 3.1 Demographics and Clinical Characteristics

A total of 1024 patients were included in the final analysis after participant screening (Fig. 1). Among them, 398 met the diagnostic criteria for MetS, yielding a prevalence of 38.9% (95% CI: 35.9%–41.9%).

As shown in Table 1, compared with the non-MetS group, patients with MetS were more likely to be male and had significantly older age and higher BMI. They were also more likely to have an illness duration of more than 5 years, clinically significant depressive symptoms, and greater illness severity as indicated by a CGI-S score of ≥4. In addition, smoking and alcohol use history were more common among patients with MetS, who also exhibited a significantly different education level distribution compared to the non-MetS group. By contrast, no significant between-group differences were observed for marital status, employment status, age at onset, or family history of psychiatric disorders.

**Table 1.** **Demographic and clinical characteristics of inpatients with schizophrenia by MetS status**.

Characteristics		Non-MetS (N = 626)	MetS (N = 398)	Total (N = 1024)	Statistic	*p*-value
					t /χ^2^	
Sex					19.21	<0.001
	Male	288 (46.0%)	239 (60.1%)	527 (51.5%)		
	Female	338 (54.0%)	159 (39.9%)	497 (48.5%)		
Age (years)		36.9 (12.6)	40.0 (11.8)	38.1 (12.4)	4.01	<0.001
BMI (kg/m^2^)		23.9 (4.1)	26.9 (4.1)	25.1 (4.3)	11.55	<0.001
Marital status					5.42	0.066
	Unmarried	332 (53.0%)	185 (46.5%)	517 (50.5%)		
	Married	192 (30.7%)	149 (37.4%)	341 (33.3%)		
	Divorced/Widowed	102 (16.3%)	64 (16.1%)	166 (16.2%)		
Employment status					1.65	0.198
	Employed	69 (11.0%)	34 (8.5%)	103 (10.1%)		
	Unemployed/Retired	557 (89.0%)	364 (91.5%)	921 (89.9%)		
Education level					7.35	0.025
	Primary school or below	137 (21.9%)	115 (28.9%)	252 (24.6%)		
	Middle and High School	385 (61.5%)	231 (58.0%)	616 (60.2%)		
	College or Above	104 (16.6%)	52 (13.1%)	156 (15.2%)		
Age of onset (years)					3.77	0.052
	<18	133 (21.2%)	65 (16.3%)	198 (19.3%)		
	≥18	493 (78.8%)	333 (83.7%)	826 (80.7%)		
Illness duration (years)					14.52	<0.001
	≤5	256 (40.9%)	116 (29.1%)	372 (36.3%)		
	>5	370 (59.1%)	282 (70.9%)	652 (63.7%)		
CGI-S score					10.05	0.002
	≤3 (Mild)	204 (32.6%)	93 (23.4%)	297 (29.0%)		
	≥4 (Moderate/Severe)	422 (67.4%)	305 (76.6%)	727 (71.0%)		
Family history of psychiatric disorders					0.50	0.481
	No	588 (93.9%)	378 (95.0%)	966 (94.3%)		
	Yes	38 (6.1%)	20 (5.0%)	58 (5.7%)		
Depressive symptoms (CDSS ≥5)					35.90	<0.001
	No	551 (88.0%)	292 (73.4%)	843 (82.3%)		
	Yes	75 (12.0%)	106 (26.6%)	181 (17.7%)		
Smoking history					28.14	<0.001
	Never	548 (87.5%)	297 (74.6%)	845 (82.5%)		
	Ever (former or current)	78 (12.5%)	101 (25.4%)	179 (17.5%)		
Alcohol use history					26.10	<0.001
	Never	587 (93.8%)	334 (83.9%)	921 (89.9%)		
	Ever (former or current)	39 (6.2%)	64 (16.1%)	103 (10.1%)		
CPZ-eq dose (mg/day)					6.95	0.031
	Low (≤200)	265 (42.3%)	159 (39.9%)	424 (41.4%)		
	Medium (201–400)	247 (39.5%)	186 (46.7%)	433 (42.3%)		
	High (>400)	114 (18.2%)	53 (13.3%)	167 (16.3%)		
Medication regimen strategy					9.24	0.010
	Non-high-risk Regimens	273 (43.6%)	144 (36.2%)	417 (40.7%)		
	High-risk Antipsychotic Regimens	290 (46.3%)	223 (56.0%)	513 (50.1%)		
	High-risk-plus-low-risk Combination Regimens	63 (10.1%)	31 (7.8%)	94 (9.2%)		

Note: Data are presented as mean (standard deviation) for continuous variables and number (percentage) for categorical variables. Group comparisons were performed using the independent-samples *t*-test or Mann–Whitney U test for continuous variables and the Pearson chi-square (χ^2^) test for categorical variables. Comparisons were performed between the MetS and non-MetS groups. Among the 179 ever smokers, 14 were former smokers and 165 were current smokers. Among the 103 ever alcohol users, 12 were former users and 91 were current users. Abbreviations: MetS, Metabolic Syndrome; BMI, Body Mass Index; CGI-S, Clinical Global Impression - Severity; CDSS, Calgary Depression Scale for Schizophrenia; CPZ-eq, chlorpromazine-equivalent.

With respect to antipsychotic treatment, the distribution of medication regimen strategies differed significantly between the MetS and non-MetS groups. Specifically, patients in the MetS group were more likely to receive high-risk regimens and less likely to receive high-risk-plus-low-risk combination regimens than those in the non-MetS group. Additionally, a significant between-group difference was observed in CPZ-eq dose categories.

### 3.2 Factors Associated With MetS Among Inpatients With Schizophrenia

The results of the multivariable logistic regression analysis are presented in Table 2. After adjustment for covariates, male sex, older age, illness duration of more than 5 years, depressive symptoms, and treatment with high-risk antipsychotic regimens remained independently associated with higher odds of MetS.

**Table 2.** **Multivariable logistic regression analysis of demographic and clinical characteristics associated with MetS**.

Variables	Coefficient	Standard error	Wald χ^2^	aOR (95% CI)	*p*-value
Age (years)	0.016	0.008	4.179	1.02 (1.00–1.03)	0.041
Onset age ≥18 years (versus <18 years)	0.102	0.201	0.256	1.11 (0.75–1.64)	0.613
Male (versus Female)	0.500	0.159	9.880	1.65 (1.21–2.25)	0.002
Duration >5 (versus ≤5 years)	0.440	0.160	7.581	1.55 (1.14–2.13)	0.006
Middle and high school (versus Primary school or below)	–0.133	0.169	0.619	0.88 (0.63–1.22)	0.431
College or above (versus Primary school or below)	–0.224	0.232	0.934	0.80 (0.51–1.26)	0.334
Married (versus Unmarried)	0.153	0.179	0.732	1.17 (0.82–1.65)	0.392
Divorced/Widowed (versus Unmarried)	–0.178	0.233	0.584	0.84 (0.53–1.32)	0.445
Depressive symptoms (versus no)	0.997	0.188	28.024	2.71 (1.87–3.92)	<0.001
CGI-S ≥4 (versus ≤3)	0.140	0.164	0.732	1.15 (0.83–1.59)	0.392
Smoking history (versus Never)	0.296	0.228	1.693	1.34 (0.86–2.10)	0.193
Alcohol use history (versus Never)	0.350	0.279	1.575	1.42 (0.82–2.45)	0.209
Medium CPZ-eq dose (versus Low)	0.055	0.152	0.132	1.06 (0.78–1.42)	0.717
High CPZ-eq dose (versus Low)	–0.255	0.217	1.382	0.78 (0.51–1.19)	0.240
High-risk antipsychotic regimens (versus Non-high-risk Regimens)	0.432	0.148	8.497	1.54 (1.15–2.06)	0.004
High-risk-plus-low-risk combination regimens (versus Non-high-risk Regimens)	0.327	0.270	1.468	1.39 (0.82–2.36)	0.226

Note: A multivariable logistic regression analysis was conducted with the presence of metabolic syndrome (MetS) as the dependent variable (the Non-MetS group served as the reference category). All variables listed in the table were mutually adjusted in the model. For categorical independent variables, the reference group is indicated in parentheses. Abbreviations: aOR, adjusted odds ratio; CI, Confidence Interval.

Specifically, male patients had a significantly greater likelihood of MetS than female patients (adjusted odds ratio [aOR] = 1.65, 95% CI: 1.21–2.25, *p* = 0.002). Increasing age was also associated with MetS (aOR = 1.02, 95% CI: 1.00–1.03, *p* = 0.041), indicating a modest increase in risk with each additional year of age. Patients with an illness duration of more than 5 years had higher odds of MetS than those with a shorter illness duration (aOR = 1.55, 95% CI: 1.14–2.13, *p* = 0.006). The presence of depressive symptoms was strongly associated with MetS (aOR = 2.71, 95% CI: 1.87–3.92, *p* < 0.001).

Regarding medication-related variables, compared with non-high-risk regimens, high-risk regimens were significantly associated with increased odds of MetS (aOR = 1.54, 95% CI: 1.15–2.06, *p* = 0.004). In contrast, high-risk-plus-low-risk combination regimens were not significantly associated with MetS (aOR = 1.39, 95% CI: 0.82–2.36, *p* = 0.226). Similarly, CPZ-eq dose was not independently associated with MetS, as neither the medium-dose nor high-dose group differed significantly from the low-dose group. In sensitivity analyses, illness duration modeled as a continuous variable remained significantly associated with MetS (aOR per 5-year increase = 1.13, 95% CI: 1.04–1.24, *p* = 0.006). No statistically significant evidence of nonlinearity was detected for age or illness duration (*p* for nonlinearity = 0.132 and 0.105, respectively). Modified Poisson regression with robust variance estimation yielded a pattern of associations consistent with the primary logistic regression analysis (**Supplementary Table 3**).

## 4. Discussion

In this multicenter cross-sectional study of 1024 inpatients with schizophrenia from two tertiary psychiatric hospitals in Central China, 38.9% met the diagnostic criteria for MetS. After multivariable adjustment, male sex, older age, illness duration of more than 5 years, clinically significant depressive symptoms, and treatment with high-risk antipsychotic regimens remained independently associated with MetS. By contrast, high-risk-plus-low-risk combination regimens and CPZ-eq dose were not independently associated with MetS. Taken together, these findings indicate that MetS was highly prevalent in the present inpatient sample and that cardiometabolic risk in this setting is shaped not only by demographic and illness-related factors, but also by depressive burden and the intrinsic metabolic liability of the prescribed antipsychotic regimen.

The observed prevalence of 38.9% is clinically noteworthy. It is higher than the pooled prevalence of 31.4% reported in a recent meta-analysis of Chinese patients with schizophrenia and also exceeds the 34.9% prevalence reported in a recent multicenter study of hospitalized patients with schizophrenia in China using the Chinese Diabetes Society criteria [5,6]. At the same time, our estimate remains slightly lower than the recent global pooled prevalence of 41.3% [4]. International evidence from both inpatient and outpatient populations has likewise shown considerable variation across treatment settings, with no consistent direction of difference between settings [3,28]. Several factors may help explain the relatively high prevalence observed in our sample. Our study focused exclusively on inpatients from tertiary psychiatric hospitals, and nearly two-thirds of participants had an illness duration of more than 5 years, indicating a predominantly chronic clinical population. In addition, differences in diagnostic criteria, patient characteristics, regional lifestyle patterns, and prescribing and hospitalization practices may contribute to variation in prevalence across studies. Clinically, the finding that nearly two in five inpatients met criteria for MetS underscores the importance of integrating cardiometabolic monitoring into routine psychiatric care.

Older age and longer illness duration were independently associated with MetS in the present study, which is consistent with prior Chinese evidence identifying age and chronic illness course as relatively robust correlates of metabolic burden in schizophrenia. These findings are biologically and clinically plausible. Over time, patients with schizophrenia may accumulate multiple cardiometabolic insults, including long-term antipsychotic exposure, reduced physical activity, poor dietary patterns, sleep and circadian disruption, and persistent stress-related neuroendocrine dysregulation. In addition, schizophrenia itself has been linked to metabolic abnormalities through inflammatory, genetic, and endocrine pathways, which may further amplify the effect of prolonged illness duration on cardiometabolic risk [2,29,30]. From a clinical standpoint, these results support a life-course approach to metabolic prevention: patients with longer illness duration and advancing age should not simply undergo the same annual screening as lower-risk patients, but may warrant more intensive and repeated metabolic surveillance.

Male sex emerged as an independent correlate of MetS in our adjusted model. This finding differs from the recent Chinese meta-analysis, which did not identify a consistent pooled sex difference [5], but is directionally compatible with broader evidence suggesting that male sex may predict greater vulnerability to antipsychotic-related metabolic dysregulation in some settings [10,31,32]. The discrepancy likely reflects the context dependence of sex effects in schizophrenia. In inpatient samples, men may have a greater accumulation of behavioral and illness-related metabolic risks over time, including more adverse lifestyle exposures and a higher burden of central adiposity, even when some of these factors do not remain independently significant after covariate adjustment. In addition, sex differences may vary according to age structure, treatment composition, and the specific diagnostic criteria used for MetS. Therefore, our findings should not be interpreted as proving a universal male disadvantage across all schizophrenia populations; rather, they suggest that in chronic inpatient settings, male sex may serve as a pragmatic marker of elevated cardiometabolic vulnerability.

An important finding of the present study is the strong independent association between depressive symptoms and MetS. Patients with clinically significant depressive symptoms had nearly threefold higher odds of MetS, even after adjustment for demographic and treatment-related factors. This result is conceptually consistent with the recent study by Avan et al. [8], which showed that MetS remained significantly associated with comorbid major depressive disorder in remitted schizophrenia after adjustment for key sociodemographic and clinical variables. Although our study evaluated depressive symptoms rather than a formal diagnosis of major depressive disorder, both findings point to a clinically meaningful link between depressive morbidity and cardiometabolic burden in schizophrenia. Several pathways may explain this association. Depressive symptoms may worsen inactivity, emotional eating, sleep disturbance, poor treatment adherence, and self-care deficits, all of which can increase metabolic risk [9,33,34]. At the same time, shared biological mechanisms, including inflammation, neuroendocrine dysregulation, and broader metabolic vulnerability, may contribute to both depressive symptoms and MetS in schizophrenia [2,35,36]. Clinically, this finding highlights that depressive symptoms in schizophrenia should not be viewed only as a psychiatric symptom cluster; they may also signal elevated metabolic risk and therefore justify parallel cardiometabolic assessment.

With regard to medication-related findings, the present study adds clinically useful nuance by moving beyond the simple distinction between antipsychotic monotherapy and polypharmacy. We found that high-risk antipsychotic regimens were independently associated with significantly greater odds of MetS, whereas high-risk-plus-low-risk combination regimens were not. This pattern suggests that metabolic associations may differ according to regimen composition rather than simply the number of antipsychotics prescribed. This is consistent with previous evidence showing that antipsychotics differ substantially in metabolic liability, with clozapine and olanzapine generally carrying the highest risk, and aripiprazole, lurasidone, and ziprasidone having more favorable metabolic profiles [10,37,38].

The pharmacological explanation for the higher MetS risk associated with high-risk regimens is plausible and supported by prior literature. Clozapine and olanzapine exhibit high affinity for histamine H1 and serotonin 5-HT2C receptors, receptor actions that are strongly implicated in hyperphagia, reduced satiety, and weight gain [39]. In addition, muscarinic M3 receptor antagonism has been linked to impaired insulin secretion and glucose dysregulation that may occur partly independently of weight gain [40]. These mechanisms provide biological plausibility for the observed association between high-risk regimens and MetS. More broadly, antipsychotic-related metabolic dysregulation appears to involve not only increased appetite and adiposity, but also direct effects on glucose homeostasis, lipid metabolism, and insulin sensitivity [41,42,43].

By contrast, the high-risk-plus-low-risk combination group did not show a statistically significant increase in MetS odds relative to the non-high-risk group. Given the wide confidence interval and the cross-sectional design, including the possibility that metabolic abnormalities had prompted prior treatment changes, this finding should be interpreted cautiously and should not be regarded as evidence of a metabolically protective effect. Nevertheless, the finding remains clinically relevant and hypothesis-generating, as the metabolic consequences of combination treatment may vary according to the metabolic liabilities of the individual agents involved. Previous studies have reported substantial heterogeneity in the relationship between antipsychotic polypharmacy and metabolic outcomes, suggesting that combination treatment should not necessarily be considered a uniform exposure [12,44]. In the present study, however, the high-risk-plus-low-risk category comprised several different combinations, and the sample was not designed to evaluate individual drug pairs. Further regimen-specific and longitudinal studies are therefore needed to clarify whether metabolic associations differ across specific combinations.

Another notable result is that CPZ-eq dose was not independently associated with MetS in the fully adjusted model, despite a significant crude association. This finding deserves separate consideration. It should not be interpreted to mean that dose is irrelevant for metabolic risk. One possible explanation is that total CPZ-eq may be too crude a metric to fully characterize the metabolic burden of antipsychotic exposure. CPZ-eq standardizes antipsychotic potency for antipsychotic efficacy, but it does not standardize affinity for receptors implicated in metabolic dysregulation, such as H1, 5-HT2C, and M3 [45]. Therefore, two regimens with similar CPZ-eq doses may differ substantially in metabolic liability because of their different receptor-binding profiles and drug-specific pharmacodynamics [40,46,47]. In addition, the dose-response relationship for metabolic adverse effects is not uniform across antipsychotics. Wu et al. (2022) [13] showed that antipsychotic-induced weight gain can follow different dose-response shapes across drugs, including plateau, ascending, and bell-shaped patterns. This suggests that once certain receptor occupancy thresholds or early metabolic changes have occurred, further increases in current dose may not translate into proportionally greater cross-sectional MetS risk. These findings suggest that the metabolic liability of specific antipsychotic regimens may contribute to MetS risk. However, the lack of association between current CPZ-eq dose and MetS should be interpreted cautiously, as current dose may not fully reflect overall antipsychotic exposure. Taken together, these considerations suggest that clinicians should be cautious about relying on CPZ-eq dose alone as a metabolic risk stratification tool.

The present study has several clinical implications. First, our findings support risk-stratified metabolic monitoring in inpatient schizophrenia care, with particular attention to male patients, older patients, those with longer illness duration, and those with depressive symptoms. Second, the results suggest that antipsychotic selection should incorporate not only psychiatric efficacy but also intrinsic metabolic liability. When high-risk agents such as clozapine or olanzapine are clinically necessary, psychiatrists should anticipate elevated MetS risk and implement proactive monitoring of waist circumference, blood pressure, fasting glucose, and lipid parameters. Third, current medication regimen and CPZ-eq dose do not capture cumulative antipsychotic exposure or prior treatment changes, and reverse causation cannot be excluded. In practice, the metabolic characteristics of individual antipsychotic regimens may provide additional information beyond current CPZ-eq dose. Finally, because depressive symptoms were strongly associated with MetS, screening for depressive burden may help identify patients who require more intensive metabolic assessment and collaborative psychiatric-medical management.

### Limitations

This study also has strengths and limitations. Among its strengths are the relatively large inpatient sample, the multicenter design, and the attempt to classify antipsychotic exposure according to metabolic-risk profile rather than only monotherapy versus polypharmacy. This pragmatic classification better reflects real-world prescribing decisions and may offer more clinically actionable information. However, several limitations should be acknowledged. First, the cross-sectional design precludes causal inference and does not establish temporality between depressive symptoms, antipsychotic exposure, and MetS. Positive and negative symptoms and extrapyramidal symptoms (EPS) were not separately assessed; despite the discriminant validity of the CDSS [17], residual symptom-related confounding cannot be excluded. Second, the sample was drawn from two tertiary psychiatric hospitals in a single city, which may limit generalizability to patients in other regions of China, secondary hospitals, outpatient settings, or community services. Third, current medication regimen and CPZ-eq dose do not capture cumulative antipsychotic exposure, prior switching patterns, or treatment duration with specific agents. Reverse causation is also possible, as patients who developed metabolic abnormalities may have undergone dose reduction, switching, or addition of a lower-metabolic-risk antipsychotic before study enrollment. Accordingly, the observed medication-related findings should be interpreted as cross-sectional associations rather than comparative metabolic effects of specific regimen strategies. Fourth, the regimen-level categories were operationally defined and necessarily encompassed heterogeneous individual antipsychotics and combinations. Although detailed descriptive information on their composition is provided, the present study was not designed or powered to estimate comparative effects of individual antipsychotics or specific combinations. Smoking and alcohol exposure were assessed categorically, without detailed quantitative measures such as smoking pack-years or amount of alcohol consumption. Finally, several potentially important confounders, including diet, physical activity, sedentary behavior, sleep, socioeconomic status, hospitalization-related factors, and concomitant medications, were not fully assessed; therefore, residual confounding, particularly for the associations with depressive symptoms and antipsychotic regimens, cannot be excluded. Future longitudinal studies should examine specific drug combinations, cumulative exposure, and changes in metabolic parameters over time to clarify the metabolic consequences of different regimen compositions.

## 5. Conclusions

In conclusion, MetS was highly prevalent in this multicenter sample of Chinese inpatients with schizophrenia, affecting nearly two in five patients. Male sex, older age, illness duration of more than 5 years, depressive symptoms, and high-risk antipsychotic regimens were independently associated with greater odds of MetS, whereas high-risk-plus-low-risk combination regimens and CPZ-eq dose were not. These findings suggest that in real-world inpatient practice, cardiometabolic risk is associated with patient vulnerability and the metabolic characteristics of prescribed antipsychotic regimens.

From a clinical perspective, the present study supports a more individualized approach to antipsychotic prescribing and metabolic surveillance. In particular, clinicians should consider incorporating regimen-based metabolic risk stratification into routine treatment planning, rather than relying solely on CPZ-eq dose or the simple presence of polypharmacy. When the use of metabolically high-risk antipsychotics is unavoidable, enhanced metabolic monitoring and early lifestyle or medical intervention should be implemented proactively. At the same time, depressive symptoms should be viewed as a clinically relevant marker for broader cardiometabolic assessment in schizophrenia. Future longitudinal and regimen-specific studies are needed, but the current findings already suggest a practical direction for care: optimize antipsychotic selection according to both psychiatric efficacy and metabolic liability, and integrate psychiatric and metabolic monitoring as a standard part of inpatient schizophrenia management.

## Data Availability

The datasets used and analysed during the current study are available from the corresponding authors on reasonable request.

## Abbreviations

MetS, Metabolic syndrome; ICD-10, International Classification of Diseases, 10th Revision; CDSS, Calgary Depression Scale for Schizophrenia; BMI, Body mass index; TG, Triglyceride; HDL-C, High-density lipoprotein cholesterol; FBG, Fasting blood glucose; SD, Standard deviation; IQRs, Interquartile ranges; aOR, adjusted odds ratio; aPR, adjusted prevalence ratio; CI, Confidence interval; CPZ-eq, Chlorpromazine-equivalent; CGI-S, Clinical Global Impression–Severity scale; EPS, extrapyramidal symptoms.

## Declaration of AI and AI-Assisted Technologies in the Writing Process

During the preparation of this work, the authors used Doubao 2.19.9 Pro exclusively to refine language fluency, enhance readability, and correct grammatical inaccuracies. No AI-generated content was incorporated into the manuscript at any stage of the writing process. The conceptual framework, methodological design, data interpretation, and scholarly conclusions remain entirely original to the authors. After using this tool/service, the authors reviewed and edited the content as needed and take full responsibility for the content of the published article.
